# The patient’s experience of participation when admitted for elective surgical procedures: an interview study

**DOI:** 10.1080/17482631.2022.2163958

**Published:** 2023-01-08

**Authors:** Maria Unbeck, Filippa Lidgren, Ella Tabbakh, Carolin Nymark

**Affiliations:** a School of Health and Welfare, Dalarna University, Falun, Sweden; b Department of Neurobiology, Care sciences and Society, Division of Nursing, Karolinska Institutet, Stockholm, Sweden; c Emergency and Reparative Medicine Theme, Department of Plastic and Craniofacial Surgery, Karolinska University Hospital, Stockholm, Sweden; d Heart, Vascular and Neuro Theme, Department of Thoracic Surgery, Karolinska University Hospital, Stockholm, Sweden; e Heart, Vascular and Neuro Theme, Department of Cardiology, Karolinska University Hospital, Stockholm, Sweden

**Keywords:** Content analysis, interviews, patient participation, person-centred care, surgery

## Abstract

**Purpose:**

To describe the patient’s experience of participation in their care when admitted for elective surgical procedures.

**Materials and methods:**

A purposive sample of 14 patients who had undergone elective surgery was included in semi-structured individual interviews at a university hospital. The data was analysed using qualitative content analysis.

**Results:**

One theme was identified: Creating a meaningful relationship to enable participation in the care, based on three categories; Abilities, willingness, and a lack of experience affect participation, A professional approach with an open communication and individualized information, and The importance of structural factors.

**Conclusions:**

To meet the patient’s individual needs and wishes regarding participation, meaningful relationships need to be created between patient and healthcare personnel. The results also indicate that the patients have insufficient knowledge about their role regarding participation. To improve patient participation, its meaning needs to be clarified individually to the patient, emphasizing the importance to be active involved in his or her own care.

## Introduction

The World Health Organization states that a crucial aspect of safety in healthcare is patient participation (World Health Organization, 2021). Patient participation increases patient satisfaction and patient safety (Fudge et al., 2008; Longtin et al., 2010; Sahlsten et al., 2008). Patient participation is a concept that may be difficult to define and no single definition exits. However, Castro et al. suggests the following definition of patient participation where “participation revolves around a patient’s rights and opportunities to influence and engage in the decision making about his care through a dialogue attuned to his preferences, protentional and combination of his experiential and the professional’s expert knowledge” (Castro et al., 2016). Patients may often experience a lack of participation in their own care and in a concept analysis patients’ participation depend on three caring science concepts, i.e., learning, caring relationship, and reciprocity. Participation could be seen as an encounter between the nurse and the patient, the caring relationship (Nilsson et al., 2019). The concept also includes sharing of knowledge and information, where the patient’s expectations and experiences are considered, and the information provided is individualized (Sahlsten et al., 2008). Patient participation may be a strategy to achieve a person-centred approach (Castro et al., 2016).

During the last few decades, the proportion of patients who want to participate in their care has increased and the expectations from the health care is to have an active patient involved in his or her own care (Sahlsten et al., 2008). Patients who undergo surgery as well as patients in cancer care, require to be more involved compared to the general population and other chronically ill patients (Chewning et al., 2012). These patients are able to make more cognizant choices regarding their care and treatment, and the healthcare’s resources can be distributed better if the healthcare personnel know what is valued by the patients (World Health Organization, 2021). Increased participation also leads to better healthcare results in several areas, e.g., patients with chronic diseases have a reduction in length of stay (Ekman et al., 2012), patients’ self-efficiency improves (Fors et al., 2015) and participation helps empower patients to handle their disease (Dudas et al., 2013) and may reduce the risk of adverse events (World Health Organization, 2013).

When undergoing general surgery, the hospital stay means a partial loss of control for the patient (Pritchard, 2009; Worster & Holmes, 2009) since the healthcare personnel have the responsibility for the patient’s vital functions. Patient participation could result in increased levels of trust, understanding and satisfaction (Shay & Lafata, 2015), and it is notable that patients who participate in their postoperative care and have responsibility for their self-care, have better outcomes such as lower pain scores, faster recovery in bowel movement, and higher resilience compared to patients who do not (Lee et al., 2018).

Elective surgery involves a waiting time for the patient from surgery decision to admission, and during this waiting time many patients experience anxiety (Rosenfeldt et al., 2011). But on the other hand, the waiting time also allows the patient to prepare for surgery (Hulzebos et al., 2012) compared to acute procedures. It is an opportunity to meet the healthcare personnel beforehand, for planned preoperative information, to understand the principles of treatment, which creates good conditions for patient participation (Aasa et al., 2013). However, there are challenges to be met, as there is limited time to involve the patient in their care. To a great extent earlier research within this field is related to a specific activity or specific areas, such as fast-track surgery (Larnebratt et al., 2019), pain-assessment (Kaptain et al., 2017), breathing (McTier et al., 2016), decisions related to the surgery (Heggland & Hausken, 2013) or participation in patients who have undergone acute surgical procedures (Lindberg et al., 2013; Malmgren et al., 2014).

To the best of our knowledge there are no studies that describe patients’ overall experience of participation when admitted for elective surgical procedures. Therefore, the purpose of this study was to describe the patient’s experience of participation in their care when admitted for elective surgical procedures.

## Materials and methods

### Design

A qualitative descriptive interview study with an inductive approach.

### Setting and sample

The data collection was carried out at two different surgical wards at a Swedish university hospital. Adult patients who had undergone elective thoracic or reconstructive plastic surgery with a length of stay of 48 hours or more and who mastered the Swedish language orally and in writing were included. Patients with a cognitive impairment or unplanned intensive care were excluded.

A purposive sample was used where a variation in age and gender was sought. The patients were identified during the hospital stay and were asked to participate by one of the authors. A total of 16 patients were invited, and 14 agreed to participate.

### Data collection

Directed by the aim of the study, an interview guide was created by the research group. The guide consisted of ten main questions with follow-up questions (Appendix). Before the data collection began, the interviewers reflected on their preunderstanding of patient participation to raise the awareness to reduce the risk of affecting the interviews. Semi-structured interviews were conducted.

Before the interview began, a modified version of a definition of patient participation (Sahlsten et al., 2008) was read to the patient to serve as an example how patient participation may be defined. The definition was

Participation can be described as active participation. In healthcare, this means an established relationship between patient and healthcare personnel, where some responsibility is handed over to the patient. Shared knowledge and information as well as a shared commitment between patient and healthcare personnel is also central to the concept.

The interviews began with the opening question “can you tell me about your overall experience of participation from your first contact with the hospital in connection with your planned surgery, until today?” Follow-up questions were asked to probe more deeply into the area. In addition, the interviews were performed consequently until saturation regarding new data was reached. Thereafter, no further interviews were conducted.

The interviews were carried out between January and February 2020 by the authors FL and ET separately. The interviewer had no established care relationship with the patient. The interviews took place in the patient´s single room and two patients had a relative present during the interview. The interviews were recorded and transcribed verbatim by the interviewer. One of the patients supplemented the content of the interview with a letter which was added to the text to be included in the analysis. The interviews lasted 15–55 (mean 33) minutes. Collected personal data, audio-recordings and their transcripts were locked up and stored with the author responsible for the study (CN).

### Data analysis

To capture the patients experiences an inductive manifest and latent content analysis followed the approach by Graneheim and Lundman (Graneheim & Lundman, 2004) was used. The transcribed text was read in its entirety and meaning units related to the aim were identified. The meaning units were condensed and labelled with a code to summarize the essence of the condensed sentences (Table 1). The generated codes were discussed and sorted into sub-categories representing the manifest content. The analysis generated three categories based on the sub-categories. Throughout the process the first authors constantly returned to the original text to ensure that the essence of the content emerged. Finally, the categories were interpreted to identify a theme that represented the latent content.

**Table I.** Examples of textual units, condensed textual units and codes.

Textual unit	Condensed textual unit	Code
What if I decide and they listen to me and it goes wrong, then it’s my own fault (laughter). No, in all seriousness, I think you know what I mean.	What if I decide and it goes wrong? Then it’s my own fault.	Fear of making the wrong decision
You want to hear a lot about that, if there was anything during the surgery that had like happened. Because it took a while before you got the answer that everything went well. Even though they said that “nah, I don’t think there was anything”, but that was just someone at the recovery room, but you don’t know that.	You want to hear if anything had happened during the surgery, it took a while before you got an answer that everything went well.	Need sanction that the surgery went well

Further, quotes were selected to illustrate the results, to verify the content of the sub-categories and to make them easier to grip (Eldh et al., 2020). Linguistic and grammatical revisions have been made because of the shift between spoken and written language and when data were translated into English.

### Rigour

Several steps were taken to increase the trustworthiness (Bowen, 2008). The interviews were conducted by two authors with several years of experience within respective clinical setting. Both interviewers listened to the recordings and the transcripts before the analysis began. During the analysis the interviewers returned to the transcribed text to avoid the analysis being affected by their pre-understanding. The analysis was reviewed on several occasions by the other authors and discussions about coding and categorization were held to increase credibility. The other two authors have long experience of elective surgical care but in other contexts which increased the variation in interpretation of the data. The collaboration between all authors, i.e., investigator triangulation, regarding the analysis further contributed to the study’s credibility and dependability (Korstjens & Moser, 2018). To further achieve credibility, we ensured to collect enough with interviews to cover variations in the data. Dependability was covered when the research team reflected on which codes and supporting quotes to be included in the categories. Reflexivity was considered by all authors throughout the process by recognizing assumptions, preconceptions, and values and how these aspects might affect the research process. To show confirmability, an example of the condensation and coding process can be seen in Table 1. Through a clear description of the steps of the study and its circumstances, conditions are created for the reader to determine transferability to other contexts (Polit & Beck, 2017).

### Ethics approval and consent to participate

The study followed the guidelines according to the declaration of Helsinki (2013). The study was approved by The Swedish Ethical Review Authority (dnr 2019–05826). The patients were informed about the study orally and in writing. Written study information was given when the patients were asked to participate, one day before the interview took place. The study information emphasized voluntary participation and guaranteed confidentiality. Each patient signed an informed consent form before inclusion. Transcribed interviews were provided with a participant-specific code number.

## Results

The demographics of the patients included in this study are shown in Table 2.

**Table II.** Patient demographics (*N* = 14).

Variables				
*Aggregated patient demographics*				
Gender, n (%)				
Female			7 (50.0)	
Male			7 (50.0)	
Age, median years (min-max)			60.5 (21–81)	
Elective admissions, n (%)			14 (100)	
Type of surgery, n (%)				
Recontructive plastic (RP)			7 (50.0)	
Thoracic (T)			7 (50.0)	
In-hospital stay at interview, median days (min-max)			4 (3–21)	
*Individual patient demographics*				
Patient number	Gender	Age	Number of in-hospital days at interview	Type of surgery
1	Male	65	4	T
2	Male	81	21	RP
3	Male	78	5	T
4	Male	57	3	RP
5	Female	78	5	T
6	Female	51	4	RP
7	Male	63	4	T
8	Female	43	5	RP
9	Female	73	4	T
10	Female	52	4	RP
11	Female	66	5	T
12	Female	42	4	RP
13	Male	21	5	T
14	Male	58	4	RP

**Figure 1.**
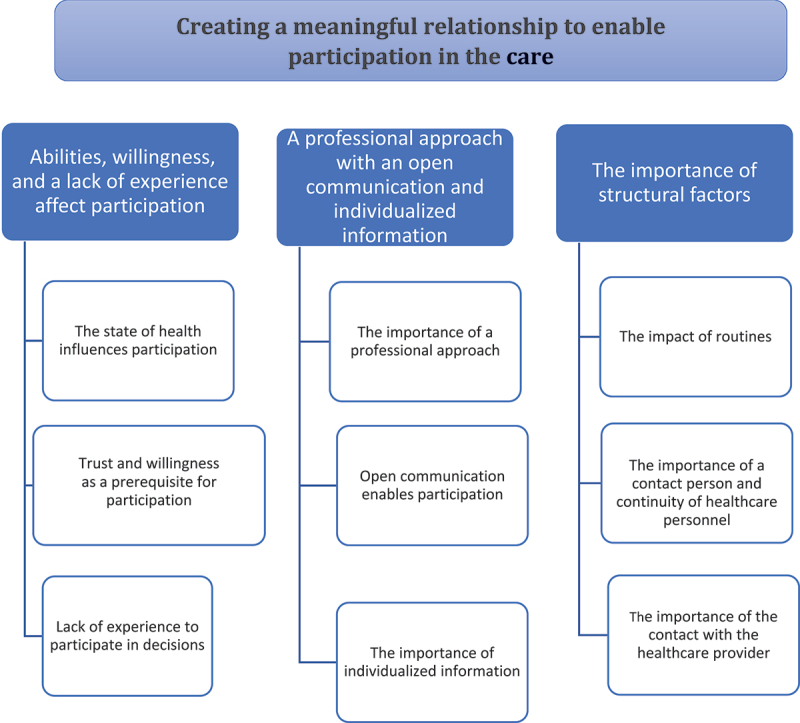
The patient’s experience of participation in their care when admitted for elective surgical procedures, described with one theme, three categories and nine sub-categories.

One main theme emerged- *creating a meaningful relationship to enable participation in the care-* based on three categories and nine sub-categories (Figure 1).

### Abilities, willingness, and lack of experiences affect participation

This category illustrates the patient’s abilities and willingness were important, which was influenced by their state of health. On the other hand, a lack of experience of being a patient could affect participation.

#### The state of health influences participation

A factor that affected patients’ experience of participation was their state of health after the surgery. Patients reported that fatigue, pain, and side effects of medications influenced their ability to participate as these could make it difficult for them to formulate questions or remember given information. There were also patients who felt no need to be involved when their health condition worsened after the surgery. They did not want to be involved in decisions or be asked questions when not feeling well. They expressed that being perceived by the health care professionals as having a normal state of health after surgery was misleading which affected their participation.

You get a little mixed up in the head, you are not at your best, you sleep, you nod off and wake up and it is even worse when you come from the recovery room. Because then I think you can be perceived as being okay, but really, you’re not, so you can’t take anything in. *(Patient with reconstructive plastic surgery)*

#### Trust and willingness as a prerequisite for participation

The patients stated that information was a prerequisite for making decisions, and a lack of these was perceived as a problem. However, being well informed did not automatically mean that patients became involved if the information did not meet their needs, nor did it mean they felt competent enough to make independent decisions. Therefore, there was a trust and willingness to completely hand over medical decisions to the healthcare personnel as most patients did not feel that they should make medical decisions regarding the surgery, even when they had the possibility.

It feels like it is the staff’s medical competence that should decide it, it’s not something I should mess with, but rather it should be arranged according to what’s best for me, that’s what I feel. There I kind of rely on those who have the competence. *(Patient with reconstructive plastic surgery)*

Through discussion with the healthcare personnel and hearing the physician’s opinion, the patients felt they were guided to the right decision. On the other hand, if information was not shared, this contributed to experiencing fear, anxiety and mistrust. To be offered alternatives regarding possible surgical techniques and type of anaesthesia, or smaller decisions like when to receive painkillers or have your meal made it easier for patients to participate in their care. Moreover, preparing checklists with questions before the ward round or using the call light to get in touch with the healthcare personnel to ask questions or express their needs, increased the feelings of being involved in decisions. Therefore, to some extent it was up to the patients to actively participate in their care. The patients described how they had been presented with alternatives in daily activities during the hospital stay and some had also been involved in decisions regarding the surgery. But then again, there were patients that did not feel they had been participating as they were not presented with any choices.

It was considered important to get the information in time but also to be informed about what was scheduled for the hospital stay as this made the patients feel calm.

I’m stuck at the X-ray department and just sitting here. And then I had been sitting there for an hour and started to think, what kind of X-ray am I really going to have? Is it’s one of those terrible ones when I go into some tube?/…/So, I built up the courage, and said, by the way what kind of X-ray am I going to do? It’s just a regular lung X-ray, just a few pictures. After that I could relax. *(Patient with thoracic surgery*)

#### Lack of experience to participate in decisions

Lack of experience of being a patient in a healthcare context could affect the ability to take the initiative to participate as patients did not know which questions could be relevant or what their needs were. Thus, patients had difficulties evaluating whether they could have been more involved than they had been. But even though they in several cases said that they wanted the healthcare personnel to guide them or make decisions for them completely, the patients still felt that they could refuse if they did not agree with the decisions or if something felt wrong.

Furthermore, the patients did not know how involved they were expected to be. On the other hand, there were reports that the patients did not have the opportunity to influence matters, e.g., the planning before the surgery even though they were motivated to do this and perceived it as important. Patients wanted to choose when the surgery would be performed, but also the method and the surgeon who would perform the surgery.

### A professional approach with an open communication and individualized information

This category illustrates that the professional approach with an open communication and individualized information was important to be treated as an individual.

#### The importance of a professional approach

The patients highlighted the importance of the professional approach of the healthcare personnel, describing situations where they were treated with respect and understanding by the healthcare personnel, and where the contact was characterized by kindness. The professional approach got the patients in a good mood and made them feel that the healthcare personnel cared about them and saw their meeting as more than a task. Several patients wanted to create a relationship with the healthcare personnel, as this enabled the conditions for the patient to be seen and treated as an individual and not just as a patient. This could include being able to talk about oneself, and the healthcare personnel taking the time to listen to the patient’s thoughts and feelings.

The fact that the patient’s individual needs were noticed by the healthcare personnel made it possible to adapt the treatment. This could mean, for example, that the healthcare personnel took additional measures when taking blood samples from a frightened patient, or made sure the patient was prepared by taking it slower and being more gentle, or it could mean spending extra time talking to a patient who had experienced something distressing. One patient illustrated how she had her needs met in the encounter with the anaesthesiologist.

And it felt quite alright at the anaesthesiologist’s to say, because I don’t know how urgent everything is, to just ask: can I have a minute to just go into myself to make myself calm? Yes of course they said, go ahead, just say when you are ready. I said, “I do this when I’m afraid”. And so, I was allowed to do it. *(Patient with thoracic surgery)*

The patients said that they trusted the healthcare personnel and the care that was given. They felt they were in a safe environment, in safe hands and that the healthcare personnel were professional and would do everything necessary for them as patients. They were also confident that the care given would be of good quality and trusted they would be invited to participate as much as possible. It was reported that when the patients felt trust in the healthcare, they had less need to participate.

They also reflected that an adverse professional approach could hinder a good care relationship and could lead to avoiding interacting with the healthcare personnel. However, the patients said that if they felt something was not working or if they were dissatisfied, the need to participate increased so they could be sure everything was done correctly. There were also patients who had experiences of being disregarded or not listened to by the healthcare personnel, which meant that the healthcare personnel did not understand their past experiences or fears. This could influence the trust and affect the relationship negatively.

#### Open communication enables participation

The patients described how open communication contributed to an interaction between them and the healthcare personnel. They also appreciated when the healthcare personnel initiated the conversations as this opened up for dialogue. A prerequisite for participation was that patients could ask questions and have their questions answered. It was described as less pressing if the patient felt well informed and previous experience of undergoing surgery made it easier to formulate questions. Several patients talked about the importance of the healthcare personnel taking their time so that they would have the opportunity to formulate questions, but also be able to get answers to the questions without time pressure. Many felt that the questions did not arise during the meeting with the healthcare personnel; instead they thought of queries after the ward round had ended and the physicians had left. This could be due to the fast ward rounds.

They come in, they see how it looks, um, everything looks good. They discuss a little with each other, though I can hear what they’re saying. And they ask how I am and if there’s anything I want to ask about. Um, and then they leave, it goes quite quickly. But I have been able to ask, I was given the opportunity if there was something/…/But it goes really fast, they are very quick so sometimes maybe you think of something afterwards. Because right at that moment you might not have time to think it through. *(Patient with recontructive plastic surgery)*

#### The importance of individualized information

Patients wished to receive clear information about the scheduling before surgery as unclear information or uncertainty was perceived as frustrating. Several patients had their surgery postponed, which they said resulted in difficulties planning their lives. After the surgery, the patients also had a desire to get a clear message about how the surgery had gone, which was not always conveyed from the surgeons who might talk about the different steps of the surgery while the patients just wanted to hear whether it had gone well or not. Patients mentioned that technical language made it difficult for them to understand the provided information. They wanted individualized information at a level that they could understand; some wanted extensive information repeated on several occasions, while others preferred limited information.

### The importance of structural factors

This category illustrate that structural factors emerged as central, such as the impact of routines that could have an influence on individualized care, having a contact person, continuity of the healthcare personnel, and the importance of the contact with the healthcare provider.

#### The impact of routines

The patients felt that areas of the care were controlled by routines and that they could not always influence these areas. They could, for example, be physical examinations and check-ups or preoperative routines. Most patients still accepted this without objection.

The patients stated that it felt safe when the healthcare personnel followed routines that they were used to, but in some situations the patients could also experience the routines as an obstacle to individual adaptation. The patients sometimes experienced the personnel as not being flexible and unwilling to deviate from the routines, despite the patients’ wishes to do so. One patient even said that he felt like being treated like a package.

It’s really easy that, that the patients, no matter how kind the staff is, get treated like packages. They are just numbers that come and go/ … /It’s a pretty dull conveyer-belt process going on. *(Patient with thoracic surgery)*

#### The importance of a contact person and continuity of healthcare personnel

Something that several patients expressed was a desire to have a contact person, as they wished to have someone to turn to before the surgery, during the hospital stay and after discharge. Several patients said that this would have made their overall experience easier as they would not have had to repeat information, and someone with an overall picture of their care could have been responsible for the entire care process. Similar comments were made regarding the continuity of the healthcare personnel, where they wanted to be cared for by the same nurse to a greater extent, even though they understood that this was not entirely possible.

#### The importance of the contact with the healthcare provider

The patients experienced that it was difficult to contact the healthcare provider before the surgery, as they did not know where and whom to turn to. One difficulty could be that when the patient was to contact the healthcare provider, this was often done by telephone and they could have to wait before being called back, and they had no possibility to influence when that would be. It could also be difficult to get in touch with the right person to answer their questions.

Once the patients made contact with the healthcare provider, they were satisfied as they perceived the healthcare personnel as helpful because they tried to meet their needs and answer questions. The patients also said that it became clearer where they should turn to for questions after a decision on surgery had been made, since contact had been established.

## Discussion

The aim of the study was to describe the patient’s experience of participation in their care when admitted for elective surgical procedures. Elective surgery involves waiting time from the decision to surgery which emphasize patients to be prepared for the surgery (Hulzebos et al., 2012) and the planned information may improve patient’s experiences of participation (Aasa et al., 2013). The findings in our study adds that a meaningful relationship enables patients to participate in their care. However, the structure of the relationship and the involvement of the patient depends partly on the patient’s abilities and on the conditions provided by the healthcare.

In the present study, a prerequisite for participation was that the healthcare personnel had time to enable an open discussion with questions and answer as many felt the questions did not arise, for example, during the rapid ward rounds. The importance of this should not be underestimated and in Sweden there is legislation that describes the patient’s rights to participate in their care, based on respect of the individual´s integrity and self-determination (Swedish Riksdag, 2014). Therefore, as far as possible, healthcare should be performed in collaboration with the patients. Leplege et al. (2007) states that an individual assessment of the patient is required where the patient’s participation in the goal formulation process should be specified. Shared information between patient and the healthcare personnel with time for discussion enables formation of common goals for the patient’s care and treatment. According to person-centred care, shared decision-making promotes increased participation for the patient (Ekman et al., 2011).

Nevertheless, to get the patients to actively participate in their care is a challenging task as participation can be problematic to define and the patients often experience an absence of participation in their care (Nilsson et al., 2019). For example, in the present study, the lack of experience of being a patient in the healthcare context affected the ability to take initiatives, and the patients had difficulties to know how to participate in their care or how involved they were expected to be. This influenced their motivation to participate, as confirmed by Tobiano et al. (2015). Therefore, they took a more passive role and perceived certain tasks as the nurses’ responsibilities. Soleimani et al. (2010) disclosed similar findings among patients with chronic diseases, as well as Ding et al. (2019) where patients with limited care experience or short length of stay, were less willing to participate in their care, and instead wanted to hand over the decisions to healthcare personnel. On the other hand, a longer length of stay may affect the patient satisfaction in a negative way (Ding et al., 2019).

Boman et al. (2018) describe that it can be overwhelming for the patient to make decisions about their care and treatment. Subsequently, one aspect of participation may be that the healthcare personnel make decisions for the patient if this is the patient’s wish. Additionally, in line with the present study, the patients expressed in several cases that they wanted to be guided in the decision-making and that it was important for the patient to speak out if they did not agree with the healthcare personnel in certain decisions, or if something felt wrong. Also, to be offered alternatives regarding, for example, surgical techniques, type of anaesthesia, and when to receive painkillers emphasized participation.

Moreover, participation increases when care is designed according to patients’ individual needs and with respect for their personal preferences and wishes (Lindberg et al., 2013; Oxelmark et al., 2018; Segevall et al., 2021). We found that being seen as individuals was facilitated by the fact that the healthcare personnel were inviting and showed that they cared about them, which meant that the patients could express their individual needs, have them met, and thus be able to adapt to the treatment. To feel trust in the care was evident, and as in line with other studies (Boman et al., 2018; Tobiano et al., 2015), the healthcare personnel’s treatment and behaviour, as well as their ability to see the patient as a person, facilitated participation. Tobiano et al. (2015) described how poor treatment from the nurses, which could include not inviting patients to participate, or engaging in task-oriented behaviour, made participation more difficult. Nurses may see participation in care as something they do on everyday basis and therefore, raising the awareness of patient participation may enhance the possibilities for the patient to participate in their care (Segevall et al., 2021).

In the present study, the patients described that information and knowledge was a prerequisite for decision-making, and a lack of these was apprehended as a problem which in turn might, contribute to a lack of perceived participation. Yet, being well informed did not automatically meant being involved if the information did not meet their needs or, if not being competent enough to make decisions. One contributing factor of lacking participation could be a limited health literacy among patients which in turn may result in less self-management (Sørensen et al., 2012). High health literacy competence means that the individual can acquire, understand, value and apply information, in order to make judgements and take decision concerning healthcare. However, to provide more information may cause patients to feel confused and powerless instead of empowering them (Ishikawa & Yano, 2008). Nevertheless, the work of improving health literacy is complex and involves not just healthcare settings but also many different areas of a community (World Health Organization, 2013).

### Limitations

The fact that not all patients were interviewed during the same postoperative day may have affected the result, as one patient had a care period that was almost five times as long as the average. The differences in length of stay could possibly affect the experience of participation, and more diversity in length of stay could have shown a more nuanced experience of participation. The patients varied in age and sex as well as treatment regimens, which ensured that a range of perceptions of the phenomenon were captured. However, interviewing patients from only one hospital may affect the transferability of the findings. Moreover, the exclusion of patients who did not speak Swedish can be seen as a weakness, as it means that the aspect of not being able to make oneself understood or to understand is missing in the results.

As described in the “rigour” section, credibility was achieved by several procedures and reflections. Another important issue to attain credibility was to interview patients who probably had experience of participation in their care (Graneheim et al., 2017). It turned out that some patients expressed that they had not been a patient in this context before and therefore had difficulties evaluating whether they could have been more involved.

Moreover, the interviewers´ definition of participation was read out loud to the patients before the interview began to achieve an understanding about the topic which also might have influenced the participants own thoughts. However, to read the same definition to all the patients strengthened their preunderstanding about “participation”.

## Conclusions

This study adds to the body of knowledge a deeper understanding of patient participation when admitted for elective surgical procedures which should be valuable for healthcare professionals in their efforts to enable participation. Patients undergoing elective surgery have individual needs for participation, and to get to them actively involved in their care is a challenging task. To meet the patient’s individual needs and wishes regarding participation, meaningful relationships need to be created between patient and healthcare personnel. The results also indicate that the patients have insufficient knowledge about their role regarding participation. To improve patient participation, its meaning needs to be clarified individually to the patient, emphasizing the importance to be active involved in his or her own care.

## Data Availability

The ethical approval for the present study does not allow us to share data.

## Appendix

1. Can you tell me about your overall experience of participation from your first contact with the hospital in connection with your planned surgery, until today?

- How does this experience compare to your expectations regarding participation?

2. How have you experienced your opportunity to participate in your care and treatment before your planned surgery?

- Were there factors which facilitated your opportunity to participate?

- Were there factors which made it difficult for you to participate?

3. During the admission conversation, how were your previous experiences, current expectations and conditions considered?

- Can you give examples?

4. How have you experienced your opportunity to participate in your care and treatment after your planned surgery?

- Were there factors which facilitated your opportunity to participate?

- Were there factors which made it difficult for you to participate?

5. In what way have you felt involved during your hospitalization?

- Can you give an example of a specific situation?

- Did you want to be more involved? So, how?

- Did you want to be less involved? So, how?

6. In what way did you feel that you did not participate during your hospitalization?

- Can you give an example of a specific situation?

- Did you want to participate more? So, how?

- Did you want to participate less? So, how?

7. If you have been part of a ward round, do you feel that you participated in the ward round discussions to the extent that you wanted?

- Were your expectations and conditions as well as questions addressed?

- Can you give examples?

- Did you want to participate more? So, how?

- Did you want to participate less? So, how?

8. How did the staff who cared for you affect your experience of participation in connection with your planned surgery?

- In what way did the staff make use of your individual conditions and abilities?

- In what way did the staff take your wishes and will into account?

- Could the staff have done it in a different way?

9. In which situations do you feel it is important to participate and why?

10. Is there anything further regarding your participation that you would like to tell me about or add?
